# Supplementary material for: Intensive care nurse managers’ experiences during the first wave of the Covid-19 pandemic: Implications for future epidemiological crises
Source: PLoS One. 2023 Aug 25;18(8):e0290722. doi: 10.1371/journal.pone.0290722 (PMC10456190; doi:10.1371/journal.pone.0290722)
Supplement: Attachment — Submitted filename: REVIEW 2023 PLOS 2.docx [file pone.0290722.s002.docx]

**University of Botswana**

**Faculty of Health Sciences**

**Report on review of a manuscript**

**Author's Names**: beata dobrowolska, PhD Aleksandra Gutysz-Wojnicka Magdalena Dziurka Patrycja Ozdoba Dorota Ozga Beata Penar-Zadarko Renata Markiewicz Agnieszka Markiewicz-Gospodarek Alvisa Palese

**Title**: Steering a boat in a storm. Qualitative analysis of ICU nurse managers’ experiences during the first wave of the Covid-19 pandemic: implications for future epidemiological crises

**1 Summary and overall impression**

The manuscript is very interesting and relevant to the health profession. The research may add to the body of knowledge about the Covid 19 pandemic which was a new strain in human history. The study is reporting the experiences of ICU nurse managers during Covid 19 pandemic. The main themes of the study were that nurse managers reported challenges with working with the unknown, methods of coping and the support received and nurse manager’s expectations.

The structure of the manuscript is well organized.

The Journal guidelines were followed by authors as required.

**2. Areas to be improved**

**2.1 Minor issues**

**Title**

The title is too long with 26 words. I suggest that it be shorted to at least 15 words

Abbreviation in the title should be avoided. The word ICU should be written in full

**Introduction section**

It is well explained, however, the authors did not state anything related to ICU and yet that is the study setting. Authors just stated the challenges and emotional impact of Covid 19 on nurse. I believe ICU was one of the settings that were critical during covid19 pandemic, therefore it is supposed to be briefly mentioned in the introduction.

Page 4- The heading ‘study design’ be tabbed to page 5 for easy flow

Page 5- the word ‘to’ be replaced with ‘of’ to read ‘contacts of nurse managers’

**2.2 Major issues**

Methods section

1. Data collection method- interview guide

The authors should briefly state where they got the questions. Were questions previously used in other studies or developed by researchers?

2. Pilot testing

It is not stated whether there was any pilot testing done. It is ideal to pilot test questions prior data collection to avoid ambiguity and make questions as clear as possible

Conclusion

The manuscript is well written.

.
