# Supplementary material for: Intensive care nurse managers’ experiences during the first wave of the Covid-19 pandemic: Implications for future epidemiological crises
Source: PLoS One. 2023 Aug 25;18(8):e0290722. doi: 10.1371/journal.pone.0290722 (PMC10456190; doi:10.1371/journal.pone.0290722)
Supplement: Attachment — Submitted filename: Response to Reviewers.docx [file pone.0290722.s004.docx]

**Response to Editor**

*Dear Editor,*

*Thank you very much for your very informative feedback and a given chance to correct our manuscript. We have carefully implemented all suggestions indicated by you and also by reviewers. All changes are introduced in a form of ‘track changes’ and below explanation is written what was changed.*

Shorten the Title and do not use abbreviations. Specify the number of participants as well as the type of study design used.

*Thank you for this important remark. We have shorten the title* *of the manuscript*. *The number of participants in the study is specified in abstract and in the manuscript body, and also the study design*.

When submitting your revision, we need you to address these additional requirements.

1. Please ensure that your manuscript meets PLOS ONE's style requirements, including those for file naming.

*Thank you for this notice. We changed file naming according to journal requirement.*

2. In the ethics statement in the Methods, you have specified that verbal consent was obtained. Please provide additional details regarding how this consent was documented and witnessed, and state whether this was approved by the IRB

*Thank you for this important remark. We added detailed information about obtaining verbal consent from study participants and how it was documented. Additionally, we also added that the protocol of the study accepted by IRB contained detailed information about the process of obtaining verbal consent.*

3. Thank you for stating the following financial disclosure:

*Thank you for this notice and apologies for misunderstanding. We did not receive any funding for this study, therefore we stated in a cover letter: “The authors received no specific funding for this work.”*

4. In your Data Availability statement, you have not specified where the minimal data set underlying the results described in your manuscript can be found.

*Thank you for this notice. We have now explained this issue in a cover letter.*

5. Please review your reference list to ensure that it is complete and correct.

*Thank you for this information. We checked our reference list. During revision we added one additional reference which is indicated in the manuscript body.*

**Response to Reviewers**

**Reviewer #1:**

*Dear Reviewer, thank you very much for the review of our manuscript and your encouraging comments!*

The manuscript authors have followed the PLOS authors guidelines. The manuscript will add to the body of knowledge about the impact of COVID 19 pandemic on the nursing profession. It is well written and the both data collection analysis procedures are relevant.

**Reviewer #2:**

*Dear Reviewer, thank you very much for valuable feedback that we received, which helped us to improve our manuscript. We corrected our work strictly following your suggestions. All changes are provided in a form of ‘track changes’ and are visible in a manuscript body. Below we also included point-by-point answer to all your remarks.*

Line 34 Suggest align number of interviewed nurse managers with the number indicated in methodology as in line 111 and 112 below is indicated that theoretical saturation achieved at 18 participants for consistency and clarity of the exact numbers where data saturation was achieved

*Thank you for this remark. We would like to apology for not being clear in stating the number of interviews with nurse managers included in final analysis. It is now corrected.*

Line 41 Suggest the training be’specific for “an in-service training” not merely training

*Thank you. We have specified this issue as suggested.*

Line 98 Suggest the study design is clearly reflected in this section and rationale for choosing the design

*Thank you for this notice. We included clear information about the study design and also justification why this study design was chosen.*

Line 102 Recheck this under measures to ensure trustworthiness in qualitative research , suggest use of dependability as reliability is more of a quantitative approach

*Thank you very much for this remark. We have corrected reliability into dependability as it should be.*

Line 130 Suggest indicate clearly how this triangulation was done to improve clarity

*Thank you for this notice. We have explained how triangulation was done in more detail.*

Line 158 Suggest this number be clarified as in line 112 saturation was achieved at 18 participants

*Thank you. We have corrected the number (n=15).*

Line 403 Suggest be consistent with use of terms, of where is referred to as subjects, nurse managers, and respondents

*Thank you. We corrected everywhere into nurse managers.*

Line 463 Suggest rephrasing of statement to improve meaning

*Thank you. We corrected indicated sentence.*

*Thank you once again for your valuable suggestions to improve our manuscript.*
